# Application of Induced Pluripotent Stem Cells for Disease Modeling and 3D Model Construction: Focus on Osteoarthritis

**DOI:** 10.3390/cells10113032

**Published:** 2021-11-05

**Authors:** Joel Jihwan Hwang, Jinhyeok Choi, Yeri Alice Rim, Yoojun Nam, Ji Hyeon Ju

**Affiliations:** 1 College of Public Health and Social Justice, Saint Louis University, St. Louis, MO 63103, USA; joel.hwang@slu.edu; 2 YiPSCELL, Inc., 39 Banpo-daero, Seocho-gu, Seoul 06579, Korea; realshine@yipscell.com (J.C.); givingtreemax@yipscell.com (Y.N.); 3 Catholic iPSC Research Center, College of Medicine, The Catholic University of Korea, Seoul 06591, Korea; llyerill0114@gmail.com; 4 Division of Rheumatology, Department of Internal Medicine, Institute of Medical Science, College of Medicine, The Catholic University of Korea, Seoul St. Mary’s Hospital, Seoul 06591, Korea

**Keywords:** osteoarthritis, induced pluripotent stem cell, disease modeling

## Abstract

Since their discovery in 2006, induced pluripotent stem cells (iPSCs) have shown promising potential, specifically because of their accessibility and plasticity. Hence, the clinical applicability of iPSCs was investigated in various fields of research. However, only a few iPSC studies pertaining to osteoarthritis (OA) have been performed so far, despite the high prevalence rate of degenerative joint disease. In this review, we discuss some of the most recent applications of iPSCs in disease modeling and the construction of 3D models in various fields, specifically focusing on osteoarthritis and OA-related conditions. Notably, we comprehensively reviewed the successful results of iPSC-derived disease models in recapitulating OA phenotypes for both OA and early-onset OA to encompass their broad etiology. Moreover, the latest publications with protocols that have used iPSCs to construct 3D models in recapitulating various conditions, particularly the OA environment, were further discussed. With the overall optimistic results seen in both fields, iPSCs are expected to be more widely used for OA disease modeling and 3D model construction, which could further expand OA drug screening, risk assessment, and therapeutic capabilities.

## 1. Introduction

**Figure 1:**
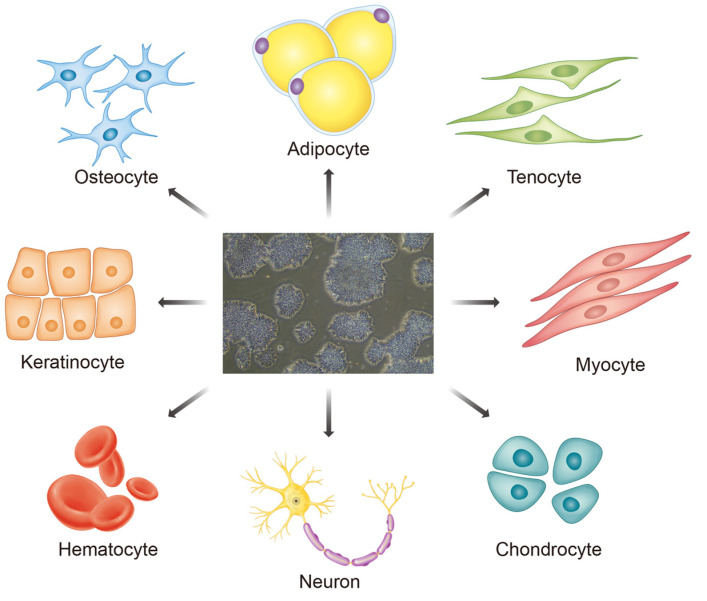
The differentiation potential of pluripotent stem cells, specifically iPSCs. Once the somatic cells are reprogrammed into iPSCs, they can be differentiated into any type of adult cell in the human body, as shown above. These iPSCs can then be used for different clinical purposes.

Pluripotent stem cells (PSCs) have promising potential in regenerative medicine because of their ability to undergo unlimited self-renewal and differentiate into any adult cell type (Figure 1) [1,2]. Four types of PSCs have been extracted from various bodily locations so far [1]. The most well-known type is the human embryonic stem cells (hESCs), which were first derived from human blastocysts by Thomson et al. in 1998 [3,4]. However, there are many ethical and political controversies surrounding hESCs that have hindered their research and use [5]. Regarding the application of hESCs, arguments regarding when human life exactly begins and what constitutes an ethical abortion have attracted political views [5,6]. Thus, the volatility associated with the research and use of hESCs has pushed for the search for alternate sources of PSCs. The other types of PSCs have their respective limitations [7]. Nuclear transfer stem cells (NTSCs) have only recently been generated from primates in 2007 and from humans in 2013 [8,9]. Furthermore, adult stem cells often involve complicated extraction procedures and have questionable clinical utility [7,10,11]. Hence, induced PSCs (iPSCs) have emerged as the most practical candidate for stem cell therapy.

## 2. Induced Pluripotent Stem Cells (iPSCs) and Their Advantages

**Figure 2:**
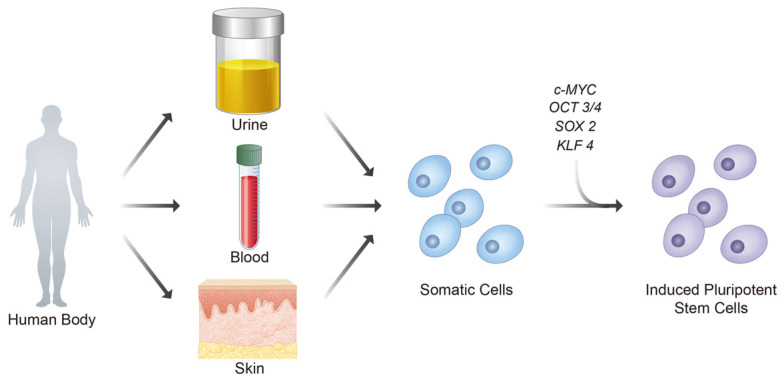
Key steps in generating iPSCs from the human samples. iPSCs can be formed by transducing OSKM factors into somatic cells derived from various locations. Notably, urine, blood, and skin are the most common samples used to collect somatic cells.

In 2006, iPSCs were first generated from murine embryonic and adult fibroblasts via the delivery of four transcription factors: *OCT3/4*, *Sox2*, *KLF4*, and *c-MYC*. They are also known as OSKM factors (Figure 2) [12]. Together, these four transcription factors reprogrammed the somatic cells to re-acquire pluripotency [13]. Using the same OSKM factors, iPSC generation was successfully replicated in adult human fibroblasts a year later [14]. As iPSCs can overcome the limitations of previous types of PSCs with additional benefits, they are expected to play a more diverse role in regenerative medicine.

For joint-related disease modeling and 3D model construction, patient-specific iPSCs surpass the limitations presented by animal models (interspecies differences, i.e., etiology) and chondrocyte culture (more complicated harvesting procedures, less realistic model, rapid dedifferentiation in vitro) [15,16]. Regarding the clinical applications of stem cells, iPSCs can be generated from several different somatic cells to avoid any complicated harvest procedures and minimize ethical conflicts in contrast to ESCs and adult stem cells. The most common somatic cells used to generate iPSCs are obtained from blood, skin, and urine (Figure 2) [7,15]. All the iPSCs generated from different somatic cells have shown similar expression of pluripotent markers, despite some variations in their potential beyond pluripotency [17]. Although more studies are required to determine the relationship between iPSC characteristics and different harvest sites, the stability and reliability of iPSCs were certainly assured in recent years [17]. Moreover, the ease of isolating iPSCs from various sites has made autologous transplantation possible in almost all cases, further reducing the risk of transplantation rejection commonly seen in non-autologous transplantations [7,18].

### Challenges in iPSCs

Despite the numerous advantages of iPSCs mentioned above, a major limitation that prevents their use is their tumorigenic potential [19,20,21]. Many studies and reviews have raised concerns regarding the development of teratomas at various body sites after iPSC administration [21,22,23,24]. Various approaches were undertaken to suppress the tumorigenic potential of iPSCs and eliminate any safety concerns. For instance, Wernig et al. reprogrammed iPSCs in the absence of *c-MYC*, which is the most oncogenic OSKM factor [19,25]. Chemical treatments via quercetin/YM155 inducing the apoptosis of undifferentiated iPSCs have also emerged as an alternative solution [26,27]. Furthermore, some studies have used immunotherapy to target the tight-junction protein Claudi-6 or SSEA-5 glycan and eliminate any undifferentiated iPSCs [28,29,30]. Nevertheless, more research is required to effectively suppress the tumorigenic potential of iPSCs so that they can be used on a larger scale.

There were also some concerns regarding the vast collection of confidential personal information, possible use of genetically modified cells, and informed consent by donors [5,31]. The use of iPSC technology certainly calls for exercising more caution because this technology holds vast amounts of private information (such as DNA) with unknown potentials and risks [31]. Hence, regulations and guidelines for iPSC studies must be developed to closely monitor clinical trials involving iPSCs [32].

## 3. Osteoarthritis

**Figure 3:**
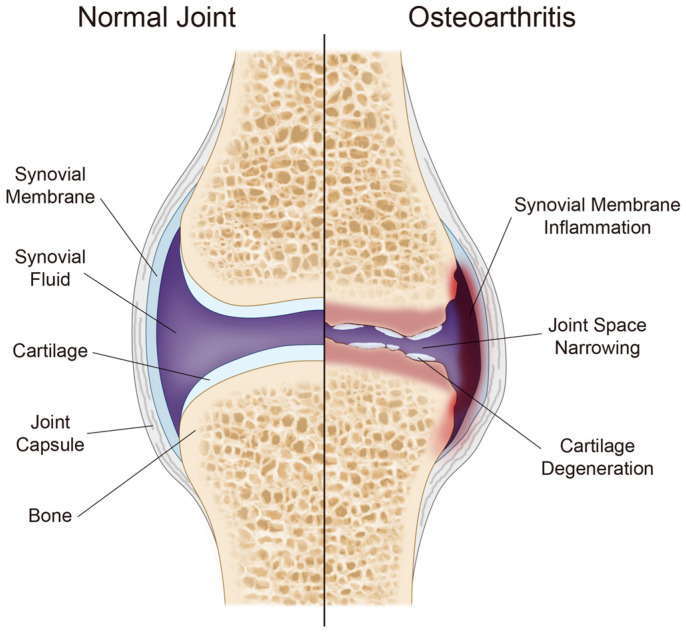
Structural image of normal and OA-diagnosed joints. OA mainly affects the cartilage, bone, and synovial membrane. Some prevalent symptoms that appear in OA-diagnosed joints are shown above.

Osteoarthritis (OA) is a degenerative joint disease whose incidence rate is expected to increase exponentially in the next decade [10,33,34]. OA is primarily characterized by joint narrowing, cartilage degradation, and synovial membrane inflammation (Figure 3) [35,36,37]. With the gradual worsening of these symptoms, joint function and movement are heavily restricted to a point where total joint replacement is required [35]. Although intra-articular (IA) injections and non-steroidal anti-inflammatory drugs (NSAIDs) have been explored for OA treatment, they face many limitations such as the short duration of action and minimal pain relief [37]. Furthermore, the complex nature of OA imposes limitations on drug availability, as they can only target specific aspects of OA, such as the inflammatory pathways, pain management, or redox signal pathways [38]. Consequently, high-risk, invasive surgical procedures are the only effective treatment for preventing OA progression [37,38]. Thus, many ongoing clinical trials are testing the safety and efficacy of various prospective OA treatments [38]. Most notably, regenerative stem cell therapies and metabolic syndrome therapies are valuable candidates that could potentially prevent or arrest OA progression without surgery [38].

OA is classified into two groups based on its etiology: primary (idiopathic and gene-dependent) or secondary (post-traumatic) [39]. However, the two groups of OA are similar in terms of disease progression; both are characterized by joint degeneration and inflammatory reactions [39,40,41]. OA prognosis is affected by various conditions, including genetic factors, age, sex, and ethnicity [10,39]. In a 2014 Research Arthritis and Articular Cartilage (RAAK) study, the genome-wide gene expression of 33 matched OA-affected and preserved cartilage sample pairs was analyzed [40]. Of the 19 genes that were expressed differently with fold-changes of 2 or more, the expression of immune response genes such as *CRLF1* and *PTGES* was upregulated, whereas that of cartilage development genes such as *COL9A1* and *CHRDL2* was downregulated [40]. More recently, Tachmazidou et al. and Boer et al. have identified additional novel OA-associated signals, such as Fibrillin 2 signal and the FGF-signaling cascade (FGFR3, FGF18, PIK3R1) in their respective genome-wide analysis [42,43]. Other risk factors for OA include obesity, physical injuries, and inflammation [10,44]. For instance, a 2016 study by Reyes et al. found a positive correlation between obesity and OA risk [45]. They concluded that individuals with grade II obesity were 4.7 times more likely to suffer from knee OA compared to individuals with a normal weight [45]. This is because the reactive oxygen species (ROS) production by OA chondrocytes stems from the mechanical overload of the joints, which further amplifies cartilage degradation [10,46]. Therefore, OA is often developed in weight-bearing joints and is mostly observed in the decreasing order of the knee, hip, and hand [33]. 

## 4. iPSC Disease Modeling

Disease modeling has greatly expanded our knowledge of pathology by recapitulating the pathophysiology and etiology of various human diseases [47,48]. For decades, animals have served as the most common experimental models of disease before human trials are performed [47]. Nevertheless, the limitations due to interspecies differences have led to high rates of translation failure between human and animal models [47,49]. Hence, constructing a disease model using human iPSCs (hiPSCs) became lucrative in 2008 when Park et al. were able to generate disease-specific iPSCs from patients diagnosed with genetic diseases (Parkinson’s disease, Huntington’s disease, Gaucher disease type III, etc.) [47,48,50,51]. By establishing a personalized disease model using the somatic cells from each patient, iPSC modeling can precisely detect any adverse side effects of potential treatments and provide a better understanding of disease phenotypes [47,50,51].

Recent advancements have considerably improved the efficacy and applicability of iPSC disease modeling. Most notably, Volpato and Webber have suggested new strategies to reduce any genetic variations by obtaining homogeneous cellular composition and establishing controls using stem cell banks [52]. Furthermore, advancements in three-dimensional organoids, microfluidic organ chips, and bioprinting (which will be discussed later) have opened new doors for iPSC disease modeling beyond the previous two-dimensional co-culturing [50].

### iPSC Disease Modeling in Various Fields

Disease modeling using iPSCs has already been used to model various neurological, cardiological, and hepatological disorders. As the earliest hESC differentiation protocols were used to generate neurons, iPSC disease models were also initially created from patients with neurological diseases [53]. Most notably, iPSC disease modeling has helped in understanding the pathogenesis of Alzheimer’s disease (AD) [53,54]. By constructing an iPSC-derived neuronal model, Israel et al. identified the relationship between proteolytic processing of amyloid-β precursor protein (APP) and phosphorylated-tau that causes neurofibrillary tangles [54,55,56]. Moreover, Wang et al. provided evidence regarding the role of apolipoprotein E4 (apoE4) in AD pathogenesis by tau phosphorylation, increased amyloid-β production, and degeneration of GABAergic neurons [57]. These discoveries and results were able to be made via hiPSC disease modeling, as apoE4 increases amyloid-beta production only in human neurons and not in those of mice, further demonstrating the benefits of iPSC disease modeling [57].

The mouse heart rate is 10 times faster than the human heart rate with key electrophysiological differences. Therefore, mouse models can be imprecise when studying human myocardial disorders [54]. In contrast, iPSC models can recapitulate cardiovascular diseases via cardiomyocyte differentiation [15,53,54]. In 2019, Zhou et al. constructed a patient-specific iPSC-derived cardiomyocyte (iPSC-CM) model of the MYL2-R58Q mutation, which is involved in severe cardiac hypertrophy [58,59]. Upon assessing the iPSC-CM model, common hypertrophic cardiomyopathy (HCM) phenotypes such as hypertrophy, myofibrillar disarray, and irregular beatings were observed, providing an optimistic outlook for understanding the pathology behind cardiovascular diseases [59].

Disease modeling of human hepatocytes is largely restricted because it requires invasive harvesting procedures and hepatocytes rapidly lose their metabolic activity in vitro [60]. However, iPSC disease modeling was able to overcome these limitations and provide a deeper understanding of hepatological diseases [54,60,61]. Numerous attempts have been made to develop an iPSC disease model of familial hypercholesterolemia (FH) [60,61,62]. The iPSC-derived FH model was confirmed to recapitulate key phenotypes such as deficiencies in low-density lipoprotein (LDL) uptake and the increased secretion of lipidated apoB-100 [62,63]. With relative success from FH iPSC modeling, cell lines with variants of FH are also being generated to cover a wide range of diseases [64].

## 5. iPSC Disease Modeling in Arthritic Diseases

Arthritic diseases were previously studied in animal models such as mice, guinea pigs, rabbits, and dogs [65,66]. However, there are several key limitations of studying arthritis in animal models [65,66,67,68,69]. As mentioned above, the distinct genetic composition of animal models presents various challenges regarding pathological characteristics that translate to human models [70,71]. Furthermore, the differences in the mechanical and clinical features, such as cartilage metabolism and antibody production, widens the gap between interspecies models for studying arthritic diseases [71,72,73,74].

iPSC-derived disease modeling for arthritic diseases avoids these complications and has proven to be an optimistic option for further exploration. To date, only a limited number of studies have been published on this topic. Kim et al. in 2018 differentiated hepatocytes from iPSCs of patients with rheumatoid arthritis (RA) [75]. These hepatocytes in 3D culture were then used to examine the effects of methotrexate (MTX)-induced hepatotoxicity that could be used for developing safe treatments in the future. Upon successful attempts by Kim et al., iPSC disease modeling for arthritic diseases has demonstrated its promising potential for future studies.

## 6. Generating iPSCs from Patients with OA

The ability to generate iPSCs from the somatic cells of OA patients was confirmed by multiple studies. In 2011, Kim et al. transduced the passage-4 synovial cells of two patients with OA using retroviruses, wherein GP2-293 cells were transfected with *pMXs-Oct-4*, *Sox2*, *Klf4*, and *c-Myc* (1:1:1:1 ratio) [76]. Similarly, Lee et al. successfully generated iPSCs from fibroblast-like synoviocytes of patients with OA [77]. Here, the synovial cells of two patients with OA were transduced with the OSKM factors via lentivirus infection. The OA iPSCs showed positive immunostaining for *Nanog*, *Oct4*, *Sox2*, *Tra-1-80*, *Tra-1-60*, and *SSEA-4*, whereas an increase in mRNA markers for *Nanog*, *Oct4*, *Sox2*, *Klf4*, and *Rex* were observed by RT-PCR. These two studies proved that iPSC colonies can be generated from the somatic cells of patients with OA and successfully laid the foundation for further research on disease modeling.

Furthermore, in 2020, Castro-Viñuelas et al. discovered that iPSC models are capable of accounting for OA-related genetic variants [78]. His team generated iPSC lines from the dermal fibroblasts of two patients with OA. At the 4th passage, the cells were reprogrammed using non-integrative Sendai RNA viruses carrying OSKM factors at 0.2–0.95% reprogramming efficiency. Notably, the presence of single nucleotide polymorphisms (SNPs) in *GDF5*, *SMAD3*, *ALDH1A2*, and *IL-1-R1* was consistent with that in the respective parental dermal fibroblasts. Thus, the different alleles were retained after reprogramming the sample from each patient, confirming patient-specific iPSC lines. This finding uncovers the vast potential of iPSC reprogramming in OA disease modeling, specifically through its ability to account for genetic variants and their respective effects on pathogenic processes. Moreover, it opens new opportunities for constructing precise, patient-specific OA disease models. In the next two Section 6.1 and Section 6.2, the recent studies that have utilized iPSC to model OA and early-onset OA will be discussed in detail and summarized in Table 1.

### 6.1. iPSC Disease Modeling in OA

In 2014, Willard et al. attempted to model OA in iPSC-derived murine cartilage to screen therapeutic agents [80]. The fibroblasts from adult C58BL/6 mice tails were first harvested and reprogrammed to iPSCs using a single doxycycline-inducible lentiviral vector carrying the OSKM factors. The generated cells were then maintained in an iPSC medium before being nucleofected with a type II collagen promoter carrying the GFP. For chondrogenic differentiation, the iPSCs were placed in a serum-free chondrogenic medium in a high-density micromass culture containing murine bone morphogenetic protein 4 and dexamethasone. The GFP-positive cells were then separated by flow cytometry and expanded in a chondrogenic medium containing fetal bovine serum and basic fibroblast growth factor. After expansion for two passages, the cells were pelleted via centrifugation and placed in a serum-free chondrogenic medium containing transforming growth factor β3 and dexamethasone until cartilage formation. The constructed iPSC cartilage, along with the native cartilage harvested from the femoral head, were treated with interleukin-1α (IL-1α) (control, 10 pg/mL, 100 pg/mL, 1 ng/mL doses for iPSC cartilage) to recapitulate the degenerative OA environment. The OA models were then used to screen five therapeutic agents (IL-4, tissue inhibitor of metalloproteinase 3, NS398, SC514, and GM6001) by assessing their tissue formation capability. Through various routes, each tested drug demonstrated its claimed effects on native tissue (i.e., matrix metalloproteinase (MMP) inhibitor GM6001 halted MMP activity). In particular, the NF-κB inhibitor SC514 considerably prevented IL-1α–mediated glycosaminoglycan (GAG) loss, and thus, was considered the most effective for tissue protection. Based on these results, this study successfully demonstrated in vitro drug screening using iPSC-derived OA models. Moreover, it demonstrated the benefits of iPSC disease modeling in terms of scalability, versatility, and sensitivity.

Lin et al. in 2019 successfully constructed human iPSC-derived OA tissue chips, further promoting the applicability of iPSC disease modeling in OA patients [83]. The hBM-MSCs from OA patients were harvested and transduced with OSKM factors into passage three via lentiviral vectors [83,85]. With the generated iPSCs, the formation of iPSC-derived MSC-like progenitor cells (iMPCs) was induced by expanding the iPSCs in mesenchymal induction medium and subsequently growing them in regular tissue culture flasks with expansion medium until passage four for further use. For constructing osteochondral tissue chips, the iMPCs were suspended in 15% mGL and placed inside micro bioreactors. The bottom of the constructs was perfused with an osteogenic medium with different combinations of VitD3, BMP7, and Dex, whereas the top of the constructs was perfused with a chondrogenic medium (containing BMP6) for 28 days. Notably, the perfusion of Dex was found to be most effective when used for the 0–14 days in a separate experiment. The chondral tissues (CHs) were created by repeating the same procedure, but only the bottom of the constructs was perfused with the cell-free mGL. These osteochondral and chondral constructs were then treated with IL-1β to recapitulate the OA conditions. After the formation of an OA disease model, its drug-screening capabilities were examined using celecoxib. Celecoxib treatment reduced the expression of catabolic factors (MMP1, 2, 9, and ADAMTS-4,5) and inflammatory factors (*IL-1*β, *IL-6*, and *COX2*) while increasing the deposition of proteoglycan and calcium without any adverse effects. Furthermore, celecoxib treatment was more effective upon administration in both the cartilage and bone rather than an IA application only to the cartilage. This could be due to the osteoprotective effects of celecoxib. In the bone tissue, celecoxib injection downregulated the expression of *IL-1*β, *IL-6*, and *OCN*, further proving its therapeutic effect. Hence, this study demonstrated the ability to construct an hiPSC OA disease model in addition to its application to study drug effects.

### 6.2. iPSC Disease Modeling in Early-Onset OA

Skeletal dysplasia is an early onset of OA characterized by osteochondral abnormalities and hindered development. In 2014, Saitta et al. recapitulated skeletal dysplasia and closely examined the molecular mechanisms underlying the cartilage disorder [79]. The fibroblasts of a patient with lethal metatropic dysplasia were used to generate iPSCs via nucleofection with nucleofector II and episomal plasmid expression vectors pCXLE-hUL, pCXLE-hSK, and pCXLE-hOCT3/4-shp53-F, and then cultured in mTeSR1 [79,86]. The iPSCs were confirmed to contain a heterozygous *TRPV4* mutation and then were differentiated into chondrocytes [79]. The chondrogenic differentiation process was induced by using various chondrogenic media (CM, CM with *BMP2*, CM with *TGFβ1*, iPSC media) where the iPSCs were cultured to confluency [79,87]. Upon Alcian blue staining, the *TRPV4*-iPSCs showed considerably fewer proteoglycans than the control, resembling the cartilage sample obtained from the patient. Moreover, the expression of *COL2A1* (forms IIA and IIB), *SOX9*, *aggrecan*, and *RUNX2* was generally downregulated in *TRPV4*-iPSCs with the upregulation of *COL1A1*, despite variations in the respective media. Through constructing an in vitro model of skeletal dysplasia, the differences in the molecular mechanisms of various signaling pathways could be more closely studied. Hence, iPSC-derived disease modeling has shown a positive outlook in understanding skeletal dysplasia and developing more effective treatment plans with specific targets, preventing early OA progression.

Yamashita et al. in 2014 also constructed a disease model of skeletal dysplasia to examine the clinical efficacy of statin treatment [81]. Dermal fibroblasts from patients with thanatophoric dysplasia type I (TD1) with heterozygous mutations in *FGFR3* were harvested. After iPSC reprogramming, the TD1-iPSCs were differentiated into chondrocytes in a chondrogenic medium. The TD1-iPSC-derived chondrocytes exhibited the disease phenotypes of skeletal dysplasia, such as the absence of GAG in Safranin O staining. Furthermore, the chondrocytes exhibited under-expression of chondrocyte markers (*Sox9*, *COL2A1*, and *ACAN*) and overexpression of *COL1A1*. Before performing further tests, the role of *FGFR3* was confirmed. In TD1-iPSCs, when mutated *FGFR3* was knocked out and transduced with a functional *FGFR3*, they were able to recover and express chondrocyte marker genes. Subsequently, different treatments were used to treat skeletal dysplasia in the disease model. An *FGFR3*-neutralizing antibody induced partial recovery of cartilage formation, as predicted from the mutated etiology. However, statins recovered cartilage formation in the TD1 disease model by mitigating the amount of phosphorylated mitogen-activated protein kinase (MAPK), which is downstream of the *FGFR3* signaling pathway. Thus, this study demonstrated the potential of using iPSC-derived disease models for drug screening to prevent the early onset of OA.

Xu et al. modeled the disease phenotypes of familial osteochondritis dissecans, a skeletal defect that signifies the early onset of severe OA [82]. In this study, both the chondrogenic differentiation and phenotypes of MSCs and iPSCs were examined. MSCs were harvested from the patient’s bone marrow and subsequently underwent chondrogenic differentiation via micropellet culture. MSC-derived chondrogenic cultures showed degradative activity, such as the absence of aggrecans upon extracellular matrix (ECM) staining and inhibition of GAG synthesis. On the other hand, iPSCs were first obtained from the patients’ fibroblasts, transfected with a retrovirus carrying OSKM factors, and finally used to generate cartilage tissues in teratomas [82,88]. Similarly, the iPSC-derived disease model of osteochondritis dissecans also produced an aggrecan-depleted ECM with densely packed cells, possibly resulting in decreased matrix production or delayed differentiation. As these similarities between the MSC-derived chondrocytes and iPSC-derived chondrocytes were confirmed, it was concluded that the iPSC-derived disease models were able to preserve the key phenotypes and provide a more accessible pathological insight.

Rim et al. recently examined the genetic characteristics of iPSC-derived OA disease models [84]. Dermal fibroblasts were harvested from a patient with radiographic early-onset finger osteoarthritis (efOA)-like condition and her healthy siblings. For generating iPSCs, OSKM factors were delivered to the fibroblasts via the Sendai virus [89]. These iPSCs then underwent chondrogenic differentiation using pellet culture to develop into osteochondral models. Hence, hiPSCs were first placed in a 1:1 mixture of E8 media and Aggrewell media to form embryonic bodies (EBs). Subsequently, the outgrown cells (OGs) were induced with the EBs in the OG induction media and then placed together in chondrogenic differentiation media to form chondrogenic pellets [84,89,90,91,92,93]. The two pellets (from efOA-like condition patient and healthy siblings) were maintained for 21 days to observe the osteochondral changes in the respective disease models. Compared with the healthy chondrogenic pellet (CP), the efOA-CP size increased drastically while exhibiting vacuole-like morphologies. The abnormal size increase could be explained by the increased expression of the hypertrophic markers *IL-6*, *MMP1*, and *MMP10*. Furthermore, other chondrogenic and hypertrophic markers, *ACAN*, *COL1A1*, and *RUNX2*, were overexpressed in efOA-CP. Interestingly, Rim et al. found evidence for establishing a relationship between the confirmed target genes (*IL-6*, *MMP1*, and *MMP10*) and IL-1β, an inflammatory cytokine. Thus, iPSC-derived disease models in OA could serve as a useful tool for understanding the pathology and genetic factors.

## 7. iPSC-Derived 3D Model Construction

Disease modeling using iPSCs has been traditionally conducted on 2D cultures because it can be easily controlled and reproduced [94,95,96]. However, this method has several limitations such as low fidelity, absence of tissue architecture, and unnatural cell morphology [94,95,96]. More importantly, 2D cultures are often unable to generate mature cells that can precisely recapitulate adult disease models [95,97]. Thus, 3D disease modeling is a possible solution with its ability to display cell–cell interactions and provide a better insight into disease mechanisms in a realistic 3D setting [98].

Broadly, there are three approaches to constructing 3D cultures: organ-on-a-chip, bioengineered scaffolds, and organoids [95]. Organ-on-a-chip was developed in 2010 by Huh et al., wherein his team constructed a biomimetic microsystem that successfully mimicked the critical functions of the alveolar–capillary interface of the human lung [99]. At a much smaller and more accessible scale, these organ-on-a-chip models process microscale fluids to mimic the human organs and tissues [100,101]. With more research performed to reduce its cost and complexities, the organ-on-a-chip model is expected to be used in large-scale preclinical trials, thereby replacing animal models [100,101,102].

Bioengineered scaffolds are the most common and approachable models used for disease modeling. Scaffolds can be constructed from a variety of materials, including hydrogels, decellularized tissue extracts, and nanofibers [95,103,104]. These 3D scaffolds closely recapitulate the ECM environment and promote tissue regeneration [105]. Notably, the 3D scaffolds can closely replicate the complexity of the human anatomy while promoting motility and intercellular communication [105]. Compared to 2D cultures, 3D scaffolds can also promote higher pluripotency and reprogramming efficiency [106]. Hence, a wider application of 3D scaffolds in disease modeling is expected. 

Organoids were acknowledged for their ability to replicate the architecture, functions, and genetic components of human organs [107]. Furthermore, research was performed to encourage their large-scale use because they are constructed through self-organization and can form complex structures without the need for a controlled environment [95,100]. The process of organoid construction begins with the activation/inhibition of the key signaling pathways to promote self-assembly [100,108,109]. Next, the media components appropriate for specific differentiation are provided. Finally, the cultures are expanded three-dimensionally by aggregating them into 3D structures or embedding them into a 3D matrix [109]. As organoids are relatively easy to construct and can recapitulate the organ architecture, they are anticipated to be used largely for drug screening and personalized medicine [100].

### 7.1. iPSC-Derived 3D Model Construction in Various Fields

The ability to generate 3D cultures from iPSCs has been used in the fields of neurology, cardiology, and hepatology. Three-dimensional cultures have provided a deeper insight into neurological disorders in understanding their pathophysiology and screening for drug toxicity. Most notably, in 2016, Raja et al. constructed brain organoids using iPSCs generated from neural tissues and were able to recapitulate the phenotypes of Alzheimer’s disease (AD), including amyloid aggregation [110,111]. Furthermore, another recent study concluded that the iPSC-derived 3D cultures can account for AD-specific genetic mutations [112]. Echoing these promising aspects, several studies have demonstrated the ability of iPSC-derived brain organoids to screen for modulators in addition to its application for therapeutic drug screening [113,114,115,116].

As 3D cultures can precisely recapitulate the human genetic background and phenotypes, various cardiac cell culture models were developed and studied [117,118]. Many cardiac conditions, such as myocardial infarction and Short QT syndrome, have already been modeled via iPSC-derived 3D cultures [119,120,121,122]. Furthermore, Takeda et al. have coated the ECM components on iPSC-derived cardiomyocytes to more efficiently construct 3D cardiac tissues [123]. Similar to iPSC-derived neural models, 3D cardiac tissues have vast potential for clinical application as they allow the control of specific parameters (i.e., oxygen content) and simulate various pathological conditions [124]. Notably, iPSC-derived 3D cardiac disease models are expected to be adopted for drug toxicity assessments [121,125,126].

Because of the limitations of 2D culture, iPSC-derived 3D cultures of hepatocytes were considered promising from their proven capabilities in other fields [127,128,129]. However, most hepatological drug screenings so far have only been performed using 2D cultures or animal models, despite 3D cultures believed to further increase the precision [130,131,132,133]. In contrast, there are abundant publications regarding the use of iPSC-derived hepatocytes to model liver diseases [61]. For instance, two studies in 2019 successfully constructed in vitro models of citrullinemia type I, steatosis, and Wolman’s disease via iPSC-derived liver organoids [134,135,136]. Once liver organoids overcome the current restrictions such as assembly requirements, they are expected to be actively used for numerous hepatological assessments and testing [137].

### 7.2. iPSC-Derived OA-Related 3D Model Construction

Since the early 2010s, attempts have been made to generate iPSCs from patients with OA and construct iPSC-derived cartilage models [76,138,139,140]. In this section, we will review recent studies that have constructed 3D chondral or osteochondral structures using iPSCs, primarily focusing on their generation procedures. As there are only a limited number of publications regarding this topic, both human and animal studies have been discussed.

To further promote the use of iPSCs in 3D cartilage model construction, more efficient protocols were developed. For instance, Yamashita et al. in 2015 generated hyaline cartilaginous tissue from human iPSCs (hiPSCs) without scaffolds [141]. The hiPSC lines were generated by transducing OSKM factors (with *Lin28* and p53) into dermal fibroblasts, dental pulp cells, and other tissues via episomal vectors [86,141]. The hiPSCs were first nucleofected with the *COL11A2-EGFP* reporter vector. Subsequently, the cells underwent chondrogenic differentiation in different cell media, first in the mesendodermal differentiation medium and then in the basal medium with chondrogenic supplementation (ascorbic acid, BMP2, *TGFβ1*, GDF5). The differentiation efficacy was confirmed by the positive GFP expression by *COL11A2-EGFP* hiPSC-derived chondrocytes and the positive staining of GAG and chondrocyte marker genes. These cartilaginous nodules were then moved to a suspension culture (with chondrogenic medium and basic fibroblast growth factor). Notably, the suspension culture contributed to eliminating non-chondrocyte cells and purifying the chondrocyte model. Furthermore, as no tumors were formed after the transplantation in the articular defects, the 3D cultures were concluded to have relatively low tumorigenic risk. In a 2021 study performed by Hall et al., iPSC-derived cartilage organoids generated from the same protocol were implanted into osteochondral defect models [142]. Therefore, the hiPSC-derived scaffold-less cartilage model is expected to increase the clinical application of 3D cultures in treating cartilage conditions.

To further investigate the flexibility of the iPSC generation protocol, Nam et al. used cord blood cell-derived hiPSCs (CBMC-hiPSCs) for CP construction [92]. The cord blood cells were initially reprogrammed with OSKM factors via the Sendai virus before their expansion to EBs [92,143]. The outgrowth cells were then induced from the EBs in gelatin-coated dishes. Subsequently, the cells were suspended in pellets with a chondrogenic differentiation medium to generate chondrogenic pellets with a 3D spheroid configuration. These pellets expressed major ECM component proteins *ACAN*, *COL2A1*, and *COMP*, and the chondrogenic marker *Sox9*. The histological characterization confirmed the presence of the ECM region with the characteristics of hyaline cartilage (decreased *COL1A1* and *COL10* expression and increased *COL2A1* expression). Based on these results, chondrogenic pellets can be generated from the CBMC-hiPSCs, which expands the iPSC sources for constructing 3D chondrogenic cultures. Furthermore, this protocol was used in a study by Rim et al., wherein the chondrogenic potential of hiPSCs from different harvest sites (skin tissue, blood, synovium, cord blood) was compared. The authors concluded that CMBC-derived chondrogenic pellets showed the maximum expression of early chondrogenic markers (*Sox9*, *Sox5*, and *Sox6*) and cartilage matrix markers (*ACAN* and *COL2A1*) [89]. 

Several studies have aimed to test the clinical applicability of the 3D iPSC-derived cultures constructed using these efficient protocols. Nguyen et al. investigated if different types of nanofibrillated cellulose (NFC) bioinks were appropriate for use in 3D bioprinting of the iPSC-derived cartilage models [144]. In this study, the two types of NFC bioinks, NFC with alginate (NFC/A) and NFC with hyaluronic acid (NFC/HA) (60/40 and 80/20 dry weight % ratio), were examined in the presence of iPSCs. These iPSCs were generated from chondrocytes via mRNA-based reprogramming and mixed with irradiated chondrocytes (iChons). The bioink consisted of iPSCs and iChons and underwent directed chondrogenic differentiation, in the order of pluripotent medium and chondrogenic medium inside the 3D-printed constructs. In bright-field microscopy, the NFC/HA bioink did not show an increase in the cell population. Both the ratios of the NFC/A bioink showed cell growth and the formation of clusters. Specifically, the 3D-bioprinted NFC/A (60/40) models showed the maximum increase in cell growth, cell viability, and decrease in the expression of *Oct4*, which is possibly tumorigenic. Moreover, the NFC/A (60/40) construct induced the formation of hyaline-like cartilaginous tissue with collagen type II expression. On the basis of these results, Nguyen et al. confirmed the applicability of NFC/A bioink in iPSC-derived cartilage construction using co-cultures with irradiated chondrocytes.

In 2020, Limraksasin et al. experimented using different protocols for generating osteochondral organoids from murine iPSCs [145]. The iPSCs were first differentiated by transducing OSK factors (without *c-Myc*) to murine fibroblasts via retroviral introduction in the growth medium. The 3D-iPSC spheres were formed in the ultra-low-attachment 24-well micro-space cell culture plates. After the sphere was constructed, trans-retinoic acid was added to promote mesenchymal precursor cells. The iPSC spheres were then maintained in shaking culture with the addition of one of the two media: osteogenic induction medium or osteogenic induction medium later replaced by chondrogenic induction medium. RT-PCR analysis showed that osteogenically induced iPSCs (OI-iPSCs) showed higher expression of osteogenic markers *Osx* and *Col1a1*, whereas osteochondrogenically induced iPSCs (OIC-iPSCs) showed higher expression of the osteogenic marker *Ocn* and chondrogenic markers *Sox9*, *Col2a1*, and *Aggrecan*. Furthermore, various staining methods showed robust mineralization and the presence of some cartilage-like tissues in the OI-iPSCs. On the other hand, OIC-iPSCs showed partial mineralization and the presence of a vast area of cartilage tissue. Hence, these findings support the use of micro-space culture and mechanical stimuli (shaking) for the formation of iPSC-derived osteochondral tissue. Moreover, the relationship between the medium used in the induction protocol and the bone/cartilage tissue ratio in these constructs can help in developing more precise control of 3D osteochondral model construction in future studies.

Similarly, O’Connor et al. successfully constructed osteochondral organoids by gradually exposing murine iPSCs to the chondrogenic and osteogenic growth factors [146]. A doxycycline-inducible lentiviral vector carrying the OSKM factors was used to generate iPSCs from mouse tail fibroblasts, which were then nucleofected with a linearized *pCOL2-EGFP-SV40-NEO* reporter plasmid. The iPSCs were then expanded with *G418*, where *G418*-resistant clones were subsequently differentiated in a micromass culture with chondrogenic media (including dexamethasone and mBMP-4). After digestion and centrifugation, the cells were separated by *GFP* expression (*GFP*+ or collagen II positive were selected). The sorted cells were then expanded in chondrogenic differentiation media (with TGF-β3) on gelatin-coated plates to induce pellet formation followed by osteochondral organoid generation by culturing the pellets in osteogenic and chondrogenic media. The pellets significantly overexpressed the chondrogenic genes *Acan*, *Col2a1*, *Prg4*, and *Sox9* in addition to osteogenic genes *Alp1*, *Bglap*, *Col1a2*, *Ibsp*, *Runx2*, and *Sp7* compared with the original iPSCs. Moreover, after staining, the presence of collagen type II, collagen type IV, and sulfated-GAGs from the chondrogenic organoids was observed, indicating successful cartilage model construction. The osteochondral organoids showed endochondral ossification with cartilaginous tissue in the center and bony calcified tissue in the surrounding area. Hence, this study used the chondrogenic and osteogenic growth factors to develop an efficient and scaffold/bioreactor-free protocol for constructing osteochondral organoids in vitro. 

Section 6.1 includes the detailed procedures for generating OA-related 3D model construction by Willard et al. and Lin et al. [80,83]. Thus, the summary of the two studies regarding their construction protocols will only be summarized in Table 2 in addition to the other studies discussed in Section 7.2.

## 8. Future Perspectives

The full potential of the clinical applications of iPSC-derived OA disease modeling and 3D cartilage model construction is yet to be discovered. Newer and more efficient protocols are still being developed in these fields. Notably, Wu et al. has targeted genes *WNT* and *MITF* to eliminate off-target differentiation and significantly increase the chondrogenic differentiation yield [147]. In the published studies discussed above, OA pathological conditions have only been recapitulated with IL-1α or IL-1β treatments; however, recapitulation with fibronectin fragments and inflammatory cytokines treatments are also expected to be used to mimic the OA environment [80,148,149,150,151]. Moreover, models that could replicate beyond the osteochondral tissues, such as the synovial membrane, can account for the broader effect of OA [83]. The advancements in both OA disease modeling and 3D cartilage model construction will be synergistic. With further research, the iPSC-derived 3D joint models could be systematically used to recapitulate OA conditions for more precise drug screening, personalized medication, and other therapeutic applications in the future [80,83].

## 9. Conclusions

In this paper, we reviewed the recent studies that reported on the use of iPSCs for OA disease modeling and 3D cartilage model construction. The iPSC-derived disease models for OA were largely successful in replicating OA phenotypes and clinical applications such as drug screening. Various protocols were generated for OA-related iPSC-derived 3D constructs. Furthermore, we examined the efficiency of different mediums, the flexibility of iPSC sources, the efficacy of bioinks, etc. [92,144,146]. Further studies however are required to be able to control the maturation of stem cell-derived chondrocytes without going through hypertrophic differentiation that leads to endochondral differentiation. The obtained results can be used as a reference for future studies to construct appropriate iPSC-derived 3D cartilage models for OA.

## Figures and Tables

**Table 1:** Summary of disease modeling in OA and early-onset OA.

Year	Reference	OA Type	iPSC Source and Reprogramming Procedure	OA Disease Model Generation Procedure	Study Objective and Results
2014s	Saitta et al. [79]	Early-onset OA (skeletal dysplasia)	Human neonatal skin fibroblasts from a patient with lethal metatropic dysplasia were nucleofected using nucleofector II and non-integrating episomal plasmid expression vectors with OSKM factors.	Heterozygous mutation of *TRPV4* confirmed in iPSC clones.	Objective: To assess the characteristics of iPSC model with a mutation in *TRPV4* (causing skeletal dysplasia). Results: The micromasses of *TRPV4*-iPSCs grown in chondrogenic differentiation conditions had lower expression of cartilage growth plate markers (*COL2A1* (IIA and IIB), *Sox9*, *Aggrecan*, *COL10A1*, *and RUNX2*), lower GAG expression, and higher expression of osteogenic differentiation marker *COL1A1*. This study successfully recapitulated skeletal dysplasia.
2014	Willard et al. [80]	Primary OA	Tail fibroblasts from adult C57BL/6 mice were transduced using single doxycycline-inducible lentiviral vector expressing mouse cDNA for OSKM factors.	The iPSC-derived cartilage model was treated with IL-1α in a serum-free chondrogenic medium for 3 days.	Objective: To construct iPSC-derived cartilage for an in vitro OA model. Results: IL-1α-treated cartilage models showed OA characteristics (increase in inflammatory and catabolic genes, decrease in tissue elastic modulus, and loss of GAG). The five therapeutic agents (IL-4, Metalloproteinase 3, NS398, SC514, and GM6001) improved OA conditions.
2014	Yamashita et al. [81]	Early-onset OA (skeletal dysplasia)	Human dermal fibroblasts from patients with thanatophoric dysplasia type I (TD1) recapitulated the disease phenotypes.	Inherited heterozygous mutation (R248C) in the *FGFR3* gene was confirmed in all samples.	Objective: To test the clinical efficacy of statin treatment in skeletal dysplasia patients. Results: The TD1 iPSCs formed abnormal chondrocyte particles that replicated TD1 phenotypes (lower GAG, *FGFR3*, cartilage matrix gene expressions). While the *FGFR3*-neutralizing antibody was induced partial recovery of cartilage formation, statin was able to successfully induce cartilage formation in TD1-iPSC-derived cartilage. This result was obtained by controlling phosphorylated MAPK production. Hence, iPSC-derived models could be used for drug screening and closely examine pathology.
2016	Xu et al. [82]	Early-onset OA (osteochondritis dissecans)	Human dermal fibroblasts from patients with familial osteochondritis dissecans were transfected using retrovirus with OSKM factors.	Inherited	Objective: To determine if cartilage models derived from BM-MSCs and iPSCs could recapitulate the phenotypes of familial osteochondritis dissecans (FOCD). Results: The FOCD-iPSC-derived cartilage displayed identical disease phenotypes in the chondrogenic cultures of primary MSCs. Both showed GAG abundance, aggrecan shortage in ECM, and aggrecan intracellular localization in early/late chondrocytes. The similarities in the disease phenotypes, such as abnormal aggrecan processing, were evident.
2019	Lin et al. [83]	Primary OA	Human bone marrow-derived MSCs from femoral heads were transduced using lentiviral vector with OSKM factors.	IL-1β was added to the chondrogenic medium that was perfused into the top of the iPSC construct during the fabrication of osteochondral tissue chips for 28 days.	Objective: To construct iPSC-derived microphysiological osteochondral tissue chips that can recapitulate OA conditions. Results: The IL-1β treatment created an OA model with a lower expression of *COL2* and *ACAN*, a decrease in the GAG, and an increase in both cartilage-degenerating enzymes and proinflammatory cytokines. The therapeutic effect of celecoxib in the OA chip model demonstrated decreased expression of catabolic and inflammatory factors in addition to its osteoprotective effect.
2021	Rim et al. [84]	Early-Onset Finger OA	Human dermal fibroblasts from a patient with radiographic early-onset finger OA-like condition (efOA-like condition) were transduced using Sendai virus with OSKM factors.	Inherited reprogrammed iPSCs contained a mutation in exon 17 of the aggrecan gene.	Objective: To construct an iPSC model of early-onset finger OA and characterize it. Results: The chondrogenic pellets from the patient with efOA-like condition displayed increase in size and vacuole-like morphologies. The abnormal size could be due to the overexpression of hypertrophic and chondrogenic markers. Hence, iPSC-derived disease models could serve as an effective tool to understand OA pathology.

**Table 2:** Summary of iPSC-derived OA-related 3D model construction.

Year	Reference	iPSC Source and Reprogramming Procedure	Cartilage Model Construction Procedure	Study Results
2014	Willard et al. [80]	Tail fibroblasts from adult C57BL/6 mice were transduced with single doxycycline-inducible lentiviral vector containing OSKM factors.	The iPSCs were placed in a high-density micromass culture with a serum-free chondrogenic medium (including BMP-4 and dexamethasone). Upon micromass digestion, the GFP+ cells were separated and expanded in a chondrogenic medium (with fetal bovine serum and basic fibroblast growth factor). These cells were then centrifuged for pellet formation before being cultured in a serum-free chondrogenic medium with *TGFβ3* and dexamethasone for cartilage model generation.	The iPSC-derived cartilage model was successfully generated and was then treated with IL-1α to recapitulate the OA environment. The OA model was used to test the clinical efficacy of current OA drugs.
2015	Yamashita et al. [141]	Human dermal fibroblasts and dental pulp were transduced using episomal factors with OSKM factors.	High-density cell colonies were first formed by culturing iPSCs in a feeder-free medium. These colonies were then cultured in a mesendodermal differentiation medium. Subsequently, the cells were put in a basal medium with various chondrogenic supplementations (combinations of ascorbic acid, BMP2, *TGFβ1*, GDF5) that generated cartilaginous nodules. Later, these models were placed in suspension culture and chondrogenic medium (for proliferation) to further be examined.	It was concluded that BMP2, *TGFβ1*, and GDF5 were needed for GFP+ cells. The suspension culture could potentially be used to separate any non-chondrocytic cells for purification purposes. This approach could be used for iPSC differentiation into scaffold-less hyaline cartilage.
2017	Nam et al. [92]	Human cord blood mononuclear cells were transduced using Sendai virus with OSKM factors.	The iPSCs underwent expansion, resuspension, and incubation to form embryoid bodies (EB). The outgrown cells from EBs were subsequently suspended in a conical tube containing a chondrogenic differentiation medium for pellet generation.	The chondrogenic pellets expressed ECM component proteins and chondrogenic markers. Moreover, the ECM region showed characteristics of hyaline cartilage. Hence, CMBC-derived iPSCs can be used to form cartilage models, which could potentially translate to therapeutic applications.
2017	Nguyen et al. [144]	Human chondrocytes underwent mRNA-based reprogramming.	The two types of bioink: NFC with alginate and NFC with hyaluronic acid were mixed with iPSCs and/or irradiated chondrocytes. Various combinations were then used for cartilage printing. Once completed, the constructs were cross-linked with either water or CaCl_2_ before rinsing and incubation. Subsequently, the constructs were placed in a pluripotent medium before undergoing differentiation in a chondrogenic medium.	The NFC/HA bioink did not show the proliferation of cells. Both ratios (80/20 and 60/40) of NFC/A bioink showed cell growth and cluster formations. NFC/A (60/40) models displayed the greatest cell growth and viability in addition to a decrease in tumorigenic expression. Moreover, the model showed the formation of hyaline-like cartilaginous tissue.
2019	Lin et al. [83]	Human bone marrow-derived MSCs from femoral heads were transduced using lentiviral vectors with OSKM factors.	The iPSCs were first differentiated into iMPCs in a mesenchymal induction medium. The iMPCs were then suspended and placed inside the microbioreactor where the constructs were perfused with a chondrogenic medium on its top side and osteogenic medium on its bottom to form osteochondral tissue chips. The chondral tissue chips were perfused with cell-free mGL solution instead of osteogenic medium.	The osteochondral and chondral tissue chips were successfully generated. The tissue chips were treated with IL-1β to recapitulate the OA environment, model OA pathology, and screen current OA drugs.
2020	Limraksasin et al. [145]	Mouse gingival fibroblasts were transduced using retrovirus with OSK factors (without *c-Myc*).	After attaining confluence, the iPSCs formed into 3D-iPSCs spheres in U-bottom-shaped microwell spots per well. The spheres were placed in an ES medium to form predominantly mesenchymal precursor cells and were later placed either in an osteogenic induction medium (OI-iPSCs) or both an osteogenic and chondrogenic medium (OIC-iPSCs) with physical shaking.	OI-iPSCs showed higher expressions of osteogenic markers: *Osx* and *Col1a1* with robust mineralization and some presence of cartilage-like tissues. OCI-iPSCs showed higher expressions of osteogenic marker *Ocn* and chondrogenic markers: *Sox9*, *COl2a1*, *Aggrecan*, and partial mineralization and strong presence of cartilage tissue. Mechanical stimuli and medium type affect the osteochondral model formation.
2020	O’Connor et al. [146]	Mouse tail fibroblasts were transduced using single doxycycline-induced lentiviral vector with OSKM factors.	iPSCs were nucleofected with linearized *pCOL2-EGFP-SV40-NEO* reporter plasmid before being expanded with G418. The G418-resistant clones were then selected to be differentiated in a micromass culture with chondrogenic media. Upon steps of centrifugation, incubation, and resuspension, GFP+ cells were separated to be expanded in chondrogenic differentiation media with TGF-β3 for pellet generation. The pellets were then cultured in chondrogenic and osteogenic media to form osteochondral organoids.	Chondrogenic pellet culture expressed chondrogenic markers and a robust cartilaginous matrix. Osteochondral organoids displayed endochondral ossification. Therefore, osteochondral organoids were able to be generated through a scaffold/bioreactor-free procedure.

## Data Availability

Data is contained with the Article.
